# The Human Fungal Pathogen *Cryptococcus neoformans* Escapes Macrophages by a Phagosome Emptying Mechanism That Is Inhibited by Arp2/3 Complex-Mediated Actin Polymerisation

**DOI:** 10.1371/journal.ppat.1001041

**Published:** 2010-08-12

**Authors:** Simon A. Johnston, Robin C. May

**Affiliations:** School of Biosciences, College of Life and Environmental Sciences, The University of Birmingham, Birmingham, United Kingdom; UMass Medical Center, United States of America

## Abstract

The lysis of infected cells by disease-causing microorganisms is an efficient but risky strategy for disseminated infection, as it exposes the pathogen to the full repertoire of the host's immune system. *Cryptococcus neoformans* is a widespread fungal pathogen that causes a fatal meningitis in HIV and other immunocompromised patients. Following intracellular growth, cryptococci are able to escape their host cells by a non-lytic expulsive mechanism that may contribute to the invasion of the central nervous system. Non-lytic escape is also exhibited by some bacterial pathogens and is likely to facilitate long-term avoidance of the host immune system during latency. Here we show that phagosomes containing intracellular cryptococci undergo repeated cycles of actin polymerisation. These actin ‘flashes’ occur in both murine and human macrophages and are dependent on classical WASP-Arp2/3 complex mediated actin filament nucleation. Three dimensional confocal imaging time lapse revealed that such flashes are highly dynamic actin cages that form around the phagosome. Using fluorescent dextran as a phagosome membrane integrity probe, we find that the non-lytic expulsion of *Cryptococcus* occurs through fusion of the phagosome and plasma membranes and that, prior to expulsion, 95% of phagosomes become permeabilised, an event that is immediately followed by an actin flash. By using pharmacological agents to modulate both actin dynamics and upstream signalling events, we show that flash occurrence is inversely related to cryptococcal expulsion, suggesting that flashes may act to temporarily inhibit expulsion from infected phagocytes. In conclusion, our data reveal the existence of a novel actin-dependent process on phagosomes containing cryptococci that acts as a potential block to expulsion of *Cryptococcus* and may have significant implications for the dissemination of, and CNS invasion by, this organism.

## Author Summary

*Cryptococcus neoformans* is fatal fungal pathogen of HIV-positive and other immunocompromised patients that causes an estimated 650 000 deaths per annum. *Cryptococcus* is able to undermine our immune system by growing within and escaping from immune cells called macrophages. In this study we describe how macrophage cells may be able to prevent this escape by forming a transient ‘cage’ of the protein actin around the intracellular pathogen. Blocking escape from within the macrophage in this way may help prevent the spread of disease around the body, especially into the brain. Thus actin flashes may represent an important host defence against diverse human pathogens.

## Introduction 

The ingestion and destruction of pathogens by phagocytes is essential to our innate immune response. Macrophages provide a front-line defence at the site of infection and, because of this, many pathogens have evolved elaborate mechanisms to subvert macrophage behaviour [1], [2], [3]. Non-lytic escape from host cells is a novel mechanism of immune avoidance first described for the fatal human fungal pathogen *Cryptococcus neoformans* by our group and others [4], [5]. Morphologically similar but molecularly distinct processes have subsequently been observed in *Chlamydia trachomatis* [6] and *Mycobacterium tuberculosis* [7], all pathogens that are characterised by a prolonged latent period. In all three cases, non-lytic exit is likely to be critical for disease dissemination, since it allows the pathogen to exit the host cell without triggering widespread inflammation due to cell lysis.

*C. neoformans* is a major cause of fatal infections of the central nervous system in HIV-positive patients and other immunocompromised individuals [8] and is an AIDS defining illness [9]. Cryptococci are able to efficiently parasitise macrophages either as a latent infection or for rapid intracellular proliferation [10]. In addition, cryptococci can be non-lytically expelled from both macrophage-like cells lines and primary mouse and human cells. This expulsion event appears similar to the ejection of indigestible *S. cerevisiae* fragments from phagosomes of the social amoeba *Dictyostelium* [11]. However a fundamental difference is that the cryptococcal expulsion requires the yeast to be alive, suggesting that it is a yeast rather than macrophage-triggered event.

The actin cytoskeleton is essential for the phagocytosis of pathogens [12] but is also commonly hijacked and subverted [13]. The human pathogens *Listeria monocytogenes*, *Rickettsia sp.*, *Shigella flexneri* and *Burkholderia pseudomallei* are all capable of being expelled from host cells by controlling the actin cytoskeleton [13]. In contrast, expulsion of *Cryptococcus* appears independent of actin polymerisation, since treatment of macrophages with the actin depolymerising drug cytochalasin D enhances, rather than blocks, expulsion [4], [5].

The interaction of *L. monocytogenes* with the host actin cytoskeleton has been intensively studied [14]. Yam and Theriot demonstrated that actin can repeatedly assemble and disassemble on phagosomes containing *Listeria* prior to their escape into the cytoplasm [15] and that this assembly may be triggered by mechanical disruption of the membrane. Recently, Liebl and Griffiths described a related phenomenon on phagosomes containing latex beads, but suggested that in this case actin flashing functioned to prevent maturation of the phagosome, by blocking fusion of lysosomal vesicles with the phagosome [16], a finding that agrees with data from *Dictyostelium* showing that filamentous actin on late-endosomal vesicles is important in preventing vesicle clustering [17].

Here we show that actin is repeatedly polymerised and depolymerised on phagosomes containing cryptococci in both macrophage-like cell lines and human primary macrophages. This process appears to inhibit cryptococcal expulsion in response to phagosome permeabilisation, suggesting that it may have an important function *in vivo* in restricting cell-to-cell spread of the pathogen.

## Results

**Figure 1:**
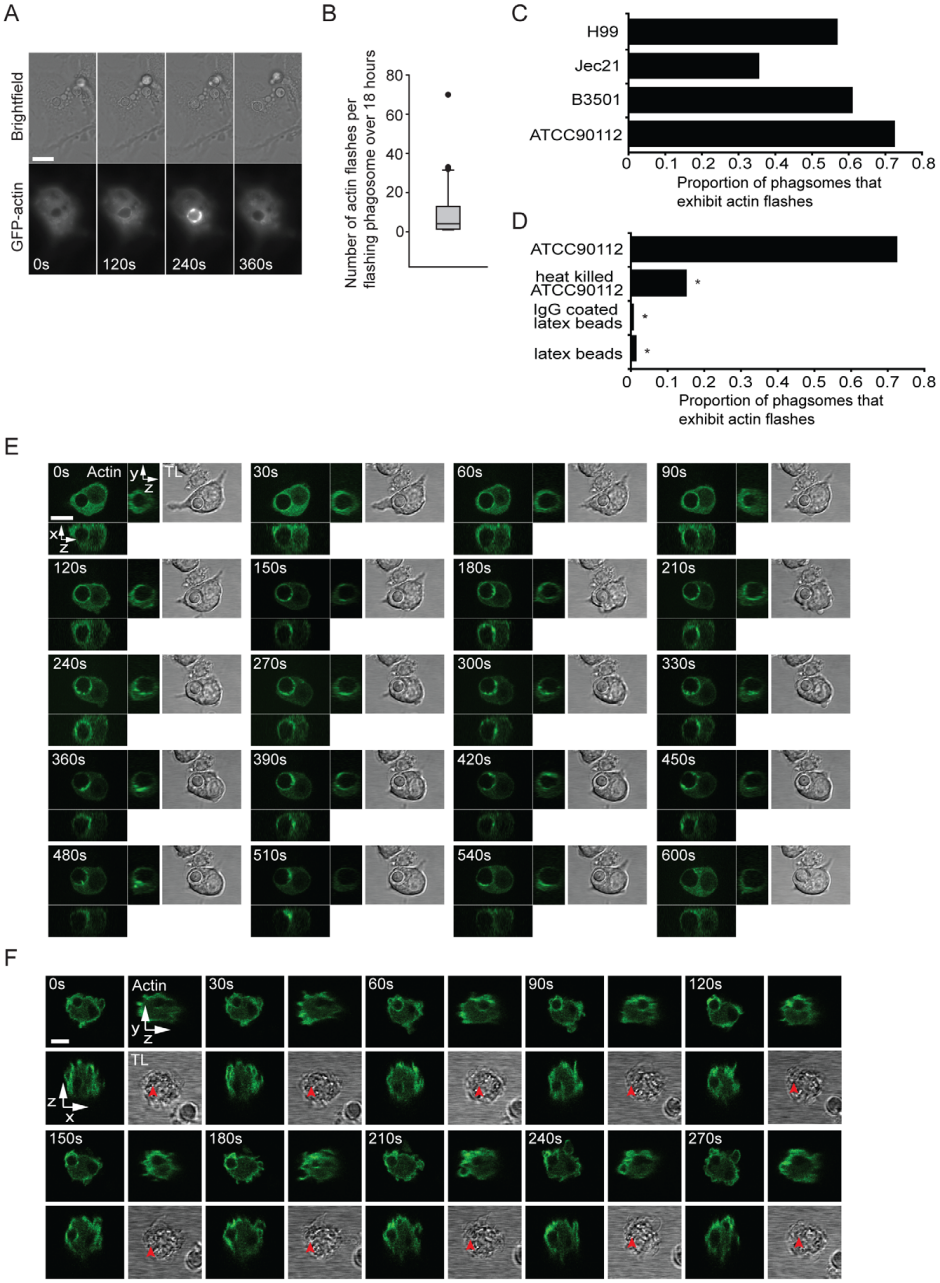
A dynamic actin coat transiently and repeatedly assembles around *Cryptococcus*-containing phagosomes. (**A**) Brightfield and actin-GFP epifluorescence images of RAW macrophage-like cell line stably expressing actin-GFP. Actin is transiently recruited to the phagosome (120s and 240s) containing a phagocytosed *Cryptococcus*. (**B**) Box plot of the number of flashes that occur on individual phagosomes. Brightfield and actin-GFP epifluorescence images of RAW macrophage-like cells stably expressing actin-GFP were captured every 2 minutes for 18 hours post phagocytosis of *Cryptococcus* and scored for actin flashes. (45 phagosomes, n = 5 experiments; excludes phagosomes that do not flash; for explanation of box dimensions see materials and methods). (**C**) The proportion of *Cryptococcus* containing phagosomes that exhibit at least one actin flash over 18 hours. Actin flashes occur on phagosomes of both serotypes of *Cryptococcus neoformans*. Serotype D: JEC21 n = 2; B3501 n = 2; Serotype A: ATCC90112 n = 6; H99 n = 3; (**D**) Comparison of the incidence of actin flashing on ATCC90112 containing phagosomes (reproduced from (C)) to heat killed ATCC90112 (n = 10, P = 2.3×10^−9^), latex beads (n = 12, P = 1.1×10^−32^) and IgG coated latex beads (n = 3 P = 8.7×10^−30^). Statistical significance is indicated by an asterisk and was tested with comparison to ATCC90112 using Fisher's exact test. (**E**) Three dimensional time lapse confocal of an actin flash around a *Cryptococcus* containing phagosome in RAW macrophage-like cell stably expressing actin-GFP. Images are representative of >100 cells observed over 5 independent experiments. (**F**) Three dimensional time lapse confocal of an actin flash around a *Cryptococcus* containing phagosome in human primary macrophage transformed with Lifeact actin sensor peptide tagged with GFP. Red arrow indicates the location of the phagosome in transmitted light images (note that it is obscured in some panels due to the large number of cortical membrane ruffles in these cells). Images are representative of 30 cells observed over 3 independent experiments. TL, transmitted light. All scale bars 10µm.

We initially used a macrophage-like cell line stably expressing actin tagged with green fluorescent protein (Raw^GFPactin^) to study the interaction between the macrophage actin cytoskeleton and phagosomes containing *Cryptococcus*. Long term time lapse imaging over 18 hours revealed that phagosomes containing cryptococci showed rapid, transient increases in actin-GFP fluorescence (Figure 1A and see Supplementary Information, Video S1) that appeared similar to actin flashes previously seen in epithelial cells infected with the Gram-positive bacterium *Listeria monocytogenes* [15] or in macrophages loaded with latex beads [16]. Time lapse imaging showed there was large variation in the frequency of actin flashing, with the median being three flashes over the duration of the experiment (18 hours) but with some cryptococcal phagosomes flashing 20 or 30 times (Figure 1B; see Supplementary information, Figure S1 for representative single-phagosome data for each of the percentiles in Figure 1B). Actin flashing occurred on all strains of *Cryptococcus neoformans* tested, including both A and D serotypes, although there was variation in the rate of flashing both within and between serotypes (Figure 1C). In contrast, heat killing of cryptococci prior to phagocytosis reduced the proportion of phagosomes that exhibited flashing over 18 hours almost 5-fold (Figure 1D). Notably, flashes were extremely rare on phagosomes containing latex beads (either unopsonised or IgG-opsonised) (Figure 1D).

Three dimensional confocal time lapse of actin flashing revealed that actin was present at multiple sites on the phagosome and was highly dynamic, moving in waves or ruffles around the phagosome (Figure 1E; see Supplementary information, Video S2 and S3).

To exclude the possibility that actin flashing was cell-line specific, we repeated this three-dimensional confocal time lapse imaging in primary human macrophages (PHM) derived from peripheral blood monocytes. To visualise actin in PHMs we transiently expressed a filamentous actin (F-actin) binding peptide (Lifeact [18]) tagged with GFP. PHMs expressing Lifeact showed similar actin flash structure and dynamics on phagosomes containing cryptococci to those observed in Raw^GFPactin^ cells (Figure 1F, see Supplementary information, Video S4).

**Figure 2:**
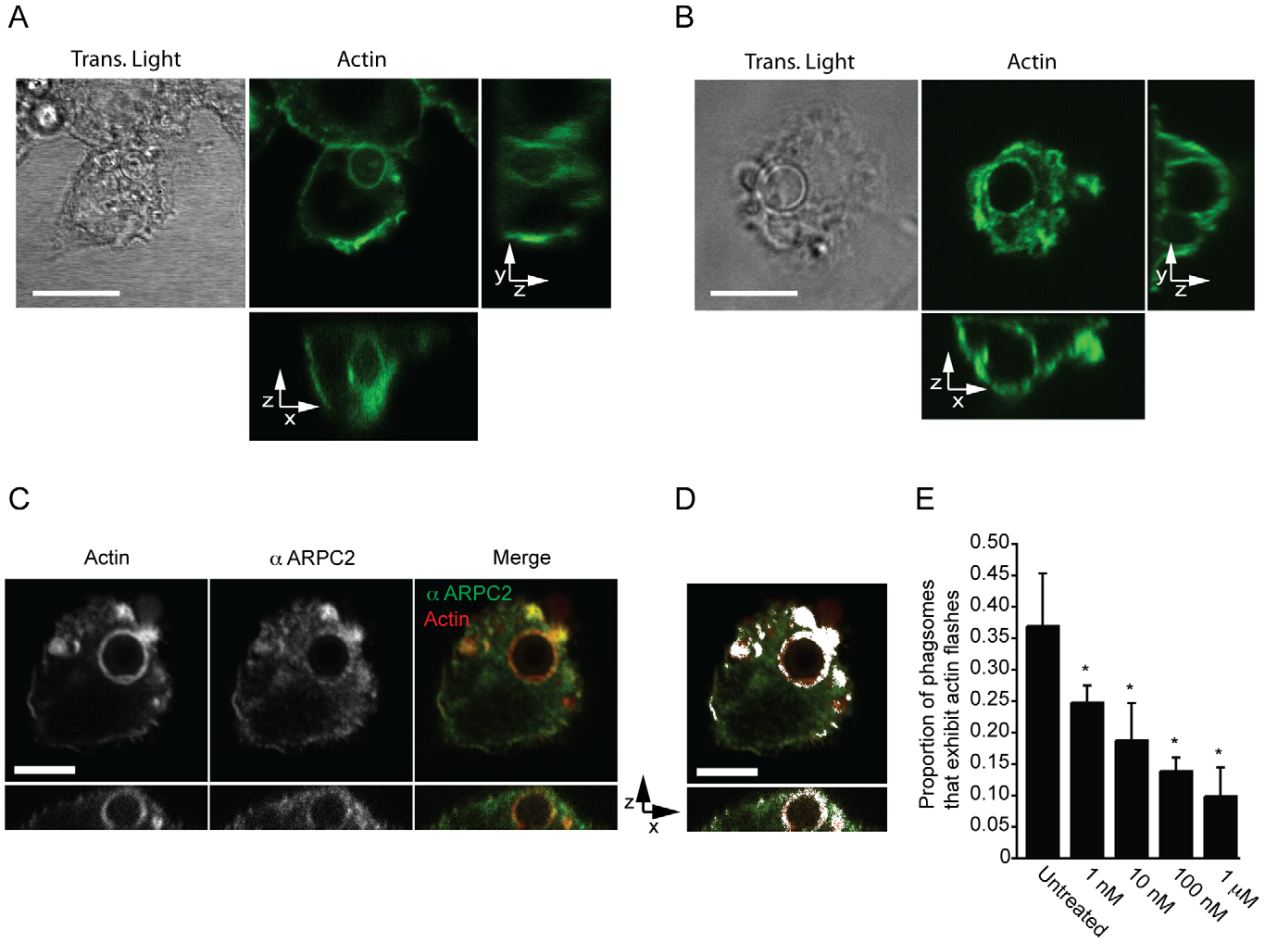
Actin flashes are generated by the Arp2/3-WASP pathway. (**A**) Transmitted and three dimensional confocal (A1R) fluorescence image of actin flash in J774 macrophage-like cells. Endogenous actin was labelled with filamentous actin binding drug phalloidin after fixation, 3 hours post phagocytosis. Scale bar 10µm. Image is representative of >100 cells observed over 3 independent experiments. (**B**) Transmitted and three dimensional confocal (A1R) fluorescence image of actin flash in human primary macrophages. Endogenous actin was labelled with filamentous actin binding drug phalloidin after fixation, 3 hours post phagocytosis. Scale bar 10µm. Image is representative of >100 cells observed over 3 independent experiments. (**C**) Three dimensional confocal (SP2) fluorescence image of a representative flashing J774 macrophage labelled for F-actin and Arp2/3 complex member ARPC2 after fixation, 3 hours post phagocytosis. Actin and Arp2/3 complex are both present on flashing phagosomes. Scale bar 5 µm. (**D**) Co-localisation of ARPC2 and F-actin, co-localised pixels are shown in white and determined as described in materials and methods. Actin and Arp2/3 complex show a high degree of co-localisation on phagosomes containing *Cryptococcus*. Scale bar 5 µm. (**E**) Proportion of phagosomes that flash in J774 macrophages following inhibition of WASP/N-WASP by wiskostatin. Cells were incubated with the indicated concentrations of wiskostatin for three hours post phagocytosis before fixation and labelling of filamentous actin. P-values are: 1nM = 0.025, 10nM = 0.011, 100nM = 2.0×10^−4^ and 1 µm = 1.3×10^−5^. Statistical significance tested with comparison to untreated using Fisher's exact test, >1000 phagosomes were scored for each concentration from a total of n = 4 experiments.

To confirm that actin flashes were formed from filamentous, rather than globular, actin we labelled infected J774 macrophage-like cells with a fluorescent F-actin binding compound, TRITC phalloidin (Figure 2A). This labelling also demonstrated that actin flashes occurred with endogenous actin in both J774 macrophage-like cells and PHMs and is not an artefact of GFP-actin or LifeAct expression (Figure 2A, B). Three-dimensional reconstruction of J774 macrophage-like cells with F-actin labelling showed that actin flashes formed an incomplete spherical cage around phagosomes (see Supplementary Information, Video S5) which agreed with the structure observed in three dimensional confocal time lapse of actin flashing in Raw^GFPactin^ cells (compare Supplementary Information, Video S6 with Video S5). Flashing phagosomes did not occupy a distinct location within the cell, but the actin cage always separated the phagosome from the cell cortex (Figure 2A, B). By specifically labelling extracellular cryptococci we confirmed that flashing phagosomes were entirely intracellular (see Supplementary Information, Figure S2).

The Arp2/3 complex, a nucleator of actin filaments, has previously been shown to be associated with *Listeria* actin flashes [15]. Similarly, antibody labelling showed that Arp2/3 complex was coincident with actin on flashing, *Cryptococcus*-containing, phagosomes and exhibits a high degree of colocalisation (Figure 2C, D). The Arp2/3 complex can be activated by the proteins WASP and N-WASP [19], which can be inhibited by the small molecule wiskostatin [20]. Scoring of actin flashes in J774 macrophage-like cells at a single time point demonstrated that by three hours post phagocytosis ∼35 percent of phagosomes are undergoing actin flashes but that low concentrations of wiskostatin inhibited actin flashes on cryptococcal phagosomes in a dose responsive manner (Figure 2E). Thus, actin flashes on cryptococcal phagosomes appear to be assembled by the activity of the Arp2/3 complex following activation by WASP family proteins.

Phagosome maturation and actin flashing has been shown to be mutually exclusive on phagosomes containing latex beads [16]. However, by using LAMP1 as a marker for mature phagosomes we found that actin flashes were present on mature phagosomes containing cryptococci (see Supplementary Information, Figure S3A). In addition, we tested whether phagosome burden (i.e. the number of individual phagosomes per macrophage) influenced actin flashing, as previously described for latex bead containing phagosomes [16]. However, flash rate did not vary between cryptococcal phagosomes in macrophages with different numbers of intracellular cryptococci indicating that phagosome burden was not positively related to actin flashing with no increase in the average number of actin flashes with >1 phagosome per cell (see Supplementary Information, Figure S3B). Thus actin flashing on cryptococcal phagosomes resembles, but is mechanistically distinct from, actin flashes previously reported on phagosomes containing latex beads.

**Figure 3:**
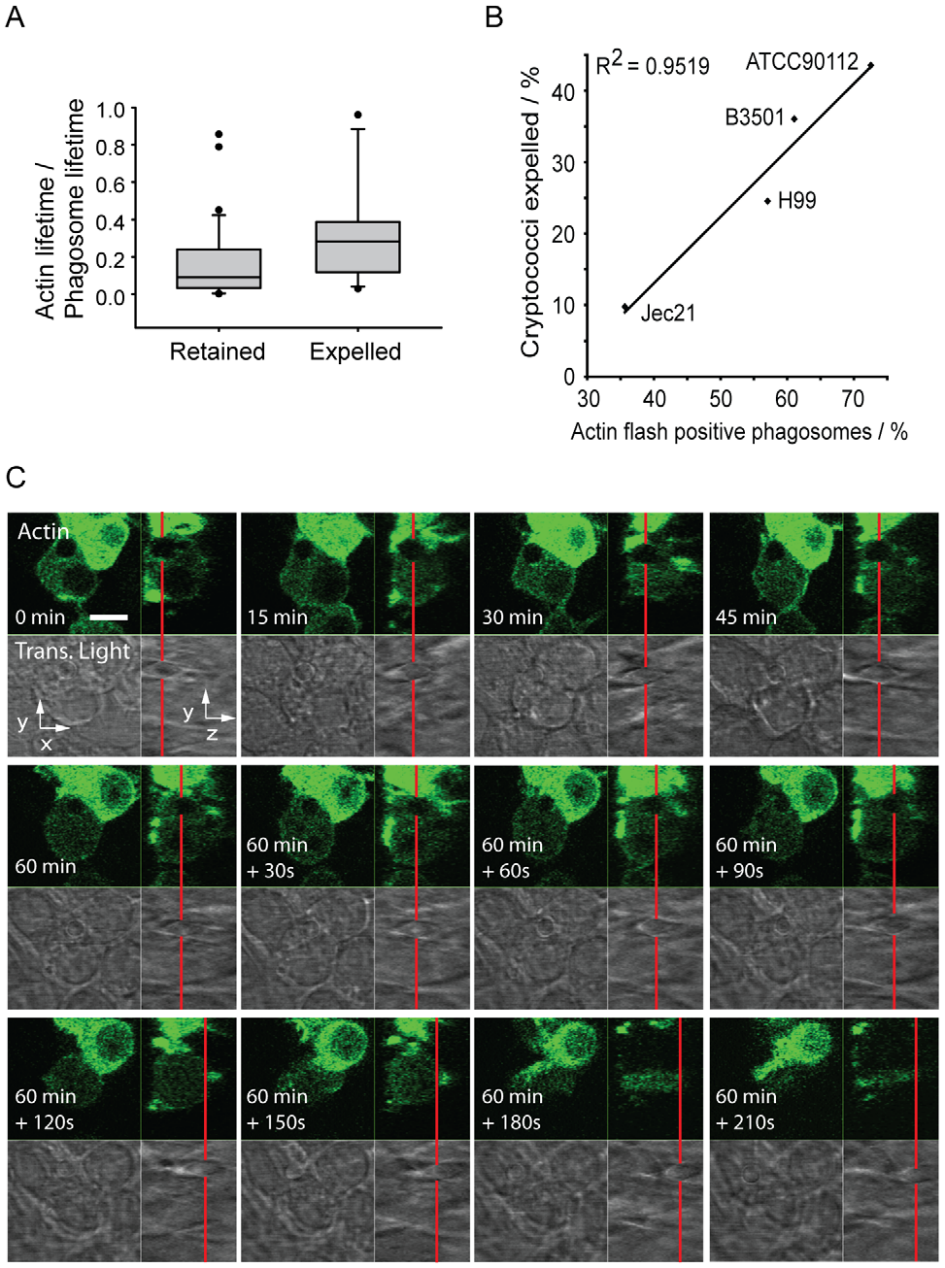
Actin flashes are positively correlated with cryptococcal expulsion but do not contribute to force generation. (**A**) Total proportion of phagosome lifetime for which an actin flash is present, comparing retained cryptococci versus those subsequently expelled. The phagosomes of expelled cryptococci spend twice the proportion of their lifetime undergoing actin flashing (mean = 0.32, median = 0.27) as those that are retained (mean = 0.16, median  = 0.08) (**B**) Four *Cryptococcus* strains were scored for the incidence of expulsion and flashing. Strains were selected to give a wide range of expulsion incidence; expulsion incidence had been previously identified for large number of strains during preliminary experiments. Both scores were performed simultaneously on the same cells. (**C**) Three dimensional time lapse confocal of the expulsion of *Cryptococcus* from RAW macrophage-like cells stably expressing actin-GFP. Red line shows the position of the phagosome in the z-axis. x,y image is the confocal z-axis slice corresponding to the position of the red line (i.e. through the centre of the phagosome). The cryptococcal phagosome is stably positioned within the cell (0–60 minutes). Two minutes prior to expulsion the phagosome moves upwards, at 60+90s can be seen partially ejected and at 60+120s has completely exited the cell. Immediately following expulsion the cryptococcal cell moves away from the macrophage. There is no increase in actin fluorescence during expulsion. Scale bar is 10 µm.

Although actin flashing on phagosomes containing cryptococci did not appear to be a barrier to phagosome maturation, we investigated the possibility that actin flashing might be a barrier to expulsion. By measuring the total time that individual phagosomes flashed as a proportion of the lifetime of the phagosome (i.e. the total time over which phagosomes were observed until expulsion or the end of the experiment) we found that the proportion of time spent flashing was significantly higher for *Cryptococcus*-containing phagosomes that were eventually expelled, as compared with those that were retained throughout the experiment (Figure 3A, p = 0.028). By correlating the previously reported variation in expulsion rate between cryptococcal strains [4], [5] to variation in incidence of actin flashing (Figure 1D) we found that the proportion of cryptococci expelled was strongly correlated with the proportion of *Cryptococcus*-containing phagosomes that exhibit actin flashes (Figure 3B, R^2^ = 0.95). However, measurement of the intensity of actin fluorescence around phagosomes containing cryptococci that were subsequently expelled established no consistent increase in actin fluorescence at the instant of expulsion, nor a regular pattern leading up to or following expulsion (see Supplementary Information, Figure S4). An example of this is shown in Figure 3C, in which a cryptococcal cell inside the macrophage (Figure 3C, 0–60 minutes) moves up towards the apical surface of the host cell (Figure 3C, 60 minutes +30s to +60s), exits the macrophage (Figure 3C, 60+90s) and is released outside of the host cell (Figure 3C, 60+120s to 210s) but at no stage shows a visible increase in actin fluorescence. Thus, transient actin polymerisation does not provide the propulsive force for cryptococcal expulsion.

**Figure 4:**
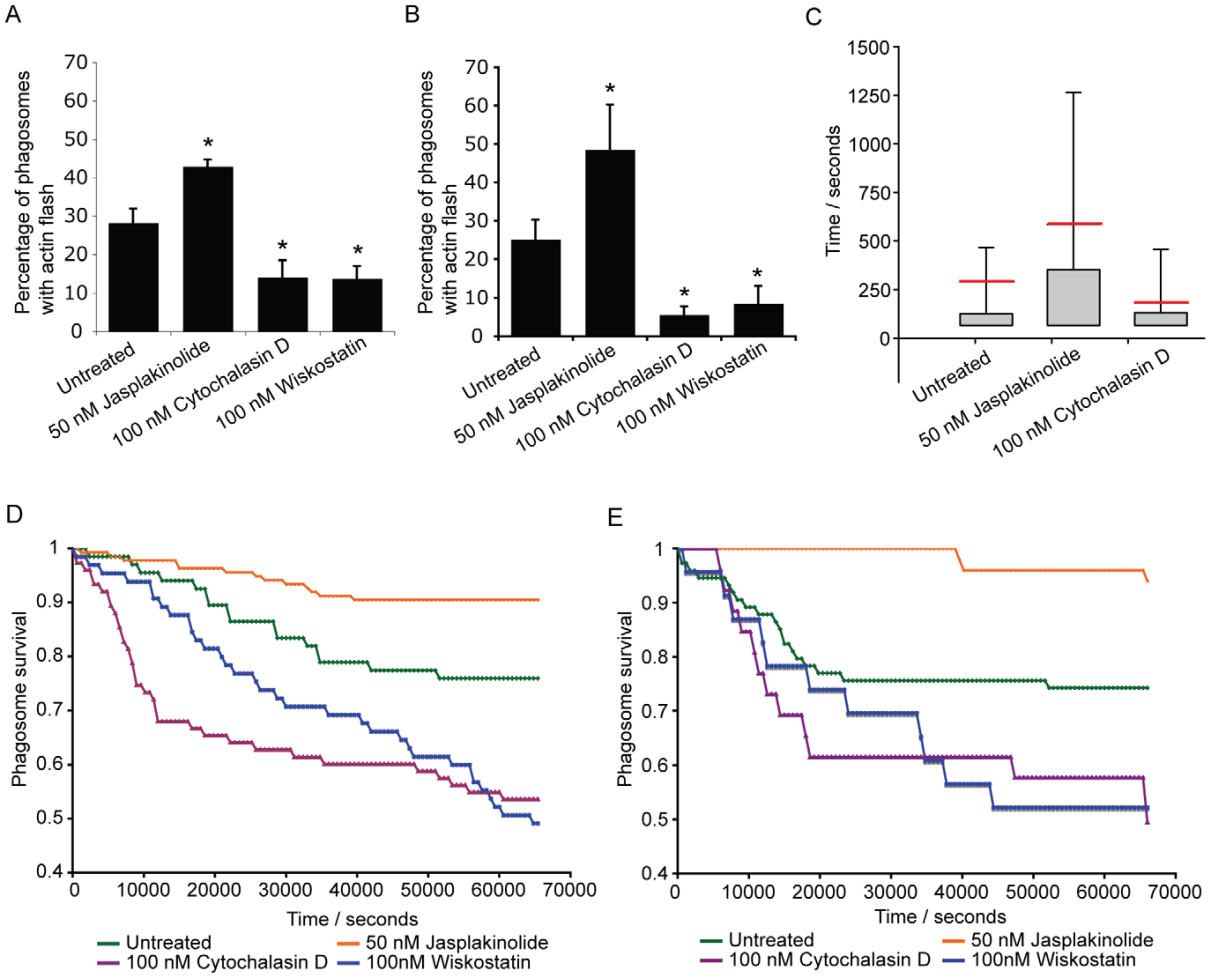
Modulation of actin flashes influences the incidence of expulsion. Actin flashes were modulated by inhibiting actin depolymerisation with 50 nM jasplakinolide, actin polymerisation with 10nM cytochalasin or WASP/N-WASP (and therefore Arp2/3 complex mediated actin polymerisation) with 100nM wiskostatin. (**A**) Percentage of phagosomes that flash in J774 macrophages with modulation of actin. Jasplakinolide, P = 1.1×10^−4^; cytochalasin D, P = 4.2×10^−8^; wiskostatin, P = 5.0×10^−10^. (**B**) Percentage of phagosomes that flash in human primary macrophages with modulation of actin. Jasplakinolide, P = 6.7×10^−5^; cytochalasin D, P = 2.9×10^−8^; wiskostatin, P = 3.4×10^−6^. (**C**) Box plots of actin flash length in RAW macrophage-like cells stably expressing actin-GFP. Horizontal red line indicates the mean. Stabilisation of actin filaments with jasplakinolide lengthens flashes while destabilising actin filaments with cytochalasin D shortens flashes. Note that the lower threshold of flash length is constrained by the frame rate of the time lapse imaging. Untreated, 68 phagosomes, n = 5 experiments; jasplakinolide, 138 phagosomes, n = 9 experiments; cytochalasin, 77 phagosomes, n = 9 experiments (**D**) Modulation of expulsion of cryptococci from RAW macrophage-like cells stably expressing actin-GFP. Expulsion of cryptococci is depicted as the proportion of phagosomes ‘at risk’ (i.e. remaining intracellular), calculated every 600s for each treatment. Influence of actin drug treatments on expulsion is the inverse of that seen with flash length. Untreated, 68 phagosomes, n = 5 experiments; jasplakinolide, 138 phagosomes, n = 9 experiments; cytochalasin, 77 phagosomes, n = 9 experiments; wiskostatin, 66 phagosomes, n = 4 experiments. (**E**) Modulation of expulsion of cryptococci from human primary macrophages. Expulsion of cryptococci depicted as the proportion of phagosomes ‘at risk’, calculated every 600s for each treatment. As with J774 macrophages, the influence of actin drug treatments on expulsion in human primary macrophages is the inverse of that seen with flash length. Untreated, 75 phagosomes, n = 3 experiments; jasplakinolide, 51 phagosomes, n = 4 experiments; cytochalasin, 27 phagosomes, n = 6 experiments; wiskostatin, 24 phagosomes, n = 3 experiments.

To further probe the role of actin flashes in cryptococcal expulsion, we manipulated flash behaviour using a series of pharmacological agents. As described above, the WASP inhibitor wiskostatin reduced actin flashing on phagosomes containing cryptococci (Figure 2E). Similarly, the actin depolymerising drug cytochalasin D reduced the number of infected cells exhibiting actin flashes in both J774 macrophage-like cells (Figure 4A) and primary human macrophages (Figure 4B). In contrast, the actin stabilising drug jasplakinolide increased the number of cells exhibiting actin flashes in both J774 macrophage-like cells (Figure 4A) and primary human macrophages (Figure 4B). The influence of cytochalasin D and jasplakinolide on the length of individual flashes was measured by time lapse imaging of Raw^GFPactin^ cells with these two different drug treatments. Treatment with cytochalasin D reduced the average duration of actin flashes by 40% (Figure 3c; mean = 173s, P = 0.004) as compared to untreated cells (Figure 3c; mean = 285s). In contrast, cells treated with jasplakinolide exhibited flashes that were on average more than twice as long as those in untreated controls (Figure 3c; mean = 576s, P = 5.3×10^−6^). The same time lapse images were then analysed for expulsion. The expulsion rates showed that cytochalasin D treatment almost doubled the incidence of expulsion whereas treatment with jasplakinolide reduced it by almost 60% (Figure 4D; untreated = 24%; jasplakinolide = 9.4%, P = 0.0068; cytochalasin D = 46%, P = 0.0046). In line with these findings, inhibiting signalling upstream of actin flashes using wiskostatin increased the incidence of expulsion to similar level seen with cytochalasin D (wiskostatin = 51%, P = 0.0012). Identical results were found when these drug treatments were repeated in human primary macrophages (Figure 4E; untreated = 26%; jasplakinolide = 6%, P = 0.0035; cytochalasin D = 51%, P = 0.043; wiskostatin = 51%, P = 0.036). These effects were due to perturbation of the macrophage, rather than cryptococcal, cytoskeleton, since pre-treatment of cryptococci, for either 2 hours before phagocytosis or during overnight culture with actin perturbing drugs prior to infection had no effect on subsequent expulsion (see Supplementary Information, Table S1). We note that cytochalasin D and wiskostatin modified the kinetics of expulsion in slightly different ways, with cytochalasin D promoting expulsion primarily within the first 3 hours whereas wiskostatin increased the rate of expulsion evenly over the time course of the experiment, perhaps reflecting differences in the mode of action of these drugs. To control for indirect effects of the actin modulating drugs on strengthening or weakening of macrophage cortical actin, we measured rates of constitutive transferrin exocytosis. Pulsed fluorescently labelled transferrin was exocytosed from Raw^GFPactin^ cells with a half-life of 21 minutes (see Supplementary Information, Figure S5A, E). In contrast to expulsion of cryptococci all three treatments slightly reduced the exocytosis of transferrin (see Supplementary Information, Figure S5B–D), contrasting with their differing effects on cryptococcal expulsion.

**Figure 5:**
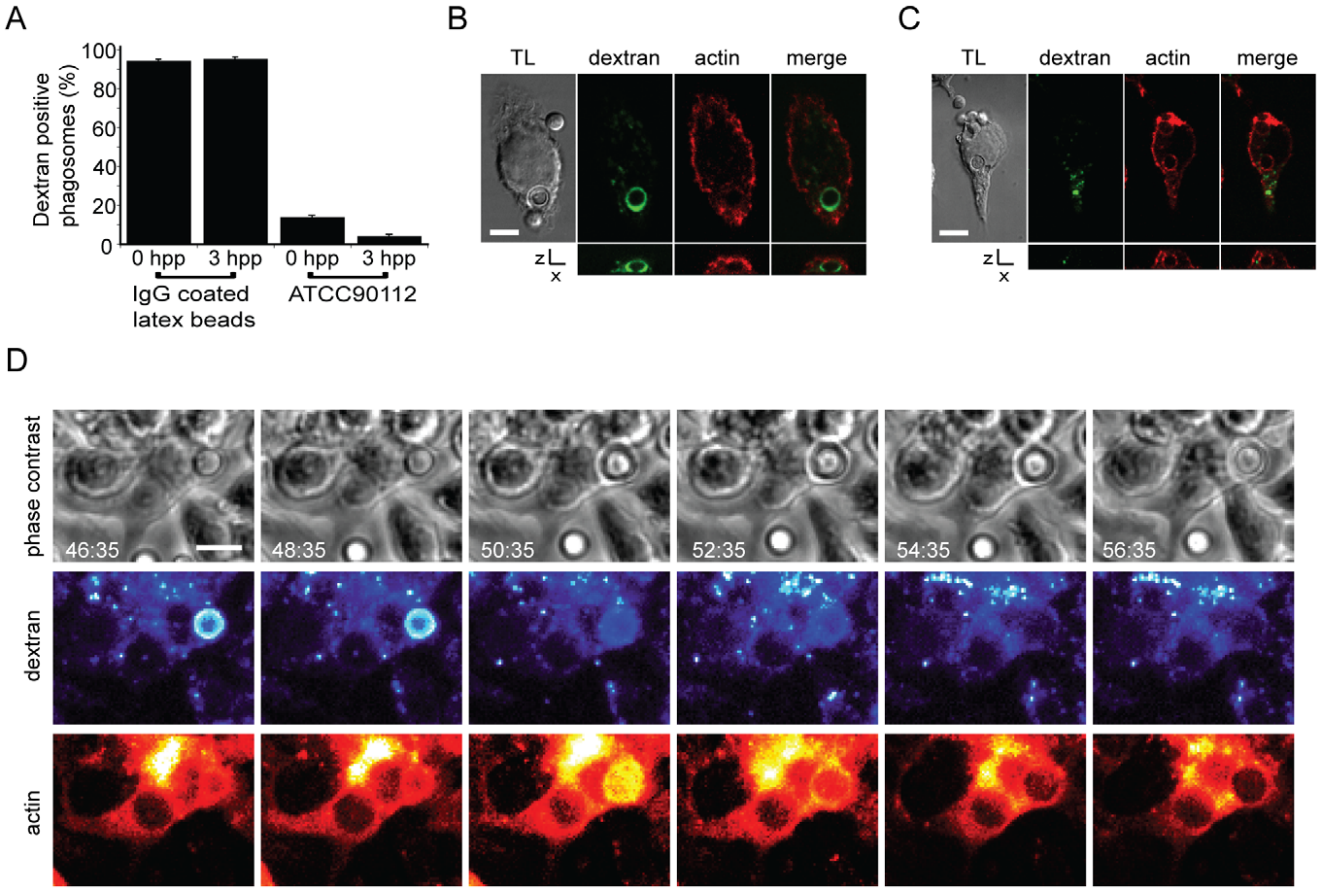
Actin flashes immediately follow rapid permeabilisation of phagosomes containing *Cryptococcus*. (**A**) J774 macrophages that had phagocytosed either IgG coated latex beads or *Cryptococcus* in the presence of FITC dextran (see figure S6) were fixed at 0 and 3 hours post phagocytosis (hpp) and scored for the presence of dextran (latex beads 0h = 450 phagosomes, 3h = 177 phagosomes; ATCC90112 0h = 1287 phagosomes, 3h = 1670 phagosomes from 3 independent experiments). (**B, C**) Dextran-positive phagosomes were never observed with actin flashes (**B**) and vice versa (**C**). Image panels are single slices from a confocal (SP2) z-stack. Transmitted light channel is labelled as TL. Scale bars 5 µm. (**D**) Time lapse phase contrast and epifluorescence images of a RAW macrophage-like cell stably expressing actin-GFP with TRITC dextran labelled, *Cryptococcus*-containing phagosomes. Phagosome permeabilisation is indicated by the loss of TRITC dextran (48:35–50:35). Note that the loss of dextran is immediately followed by an actin flash. Scale bar 5 µm.

Given this correlation between modulation of actin flashes and specific modulation of expulsion, we tested a possible role for phagosome membrane permeabilisation in actin flashing. As previously described [21], addition of fluorescently labelled dextran during phagocytosis of cryptococci provides a distinct, stable label of phagosome integrity (see Supplementary Information, Figure S6A, B and Video S7). When a cryptococcal cell was expelled there was a sudden and complete loss of dextran fluorescence from the macrophage and no fluorescence associated with the expelled cryptococci (see Supplementary Information, Figure S6C, Video S8), indicating that expulsion occurs via a membrane/membrane fusion mechanism. Membrane labelling of fixed J774 macrophage-like cells showed that in 58% of phagosomes undergoing an actin flash the phagosome membrane appears to be incomplete (26 three-dimensional confocal reconstructions of phagosomes with actin flashes from three independent experiments). Although dextran labelling was very stable in some cases, the vast majority of cryptococcal phagosomes lose dextran soon after phagocytosis (Figure 5A), suggesting that they are rapidly permeabilised. In contrast, phagosomes containing latex beads showed stable dextran labelling over the same time period (Figure 5A). By scoring phagosomes (1883 phagosomes, n = 4 experiments) for the presence of dextran together with actin flashes, we observed that actin flashes are only ever observed on dextran-negative (i.e. permeabilised) phagosomes (Figure 5A,B; censoring phagosomes negative for both dextran and actin flashes, dextran positive = 36.9%, flash positive = 63.1%, dextran and flash positive = 0%, P = 0.0046). Time lapse imaging of dextran positive phagosomes demonstrated that when dextran was lost an actin flash immediately followed (Figure 5C and Supplementary Information, Video S9). Thus phagosome permeabilisation appears to provide a stimulus for actin flash assembly.

## Discussion

The non-lytic expulsion of cryptococci is a poorly understood phenomenon that may have significant clinical implications, for example in understanding Trojan horse mediated invasion of the CNS [22], [23], [24]. Here we demonstrate that transient actin flashes, previously reported on phagosomes containing *Listeria* or latex beads [15], [16], also occur on phagosomes containing *Cryptococcus*. These flashes occur in both human and murine macrophages and across a range of cryptococcal strains. Using three dimensional confocal time lapse we show that actin polymerises at multiple sites around the phagosome, developing into waves or ruffles which in turn form a cage around the phagosome. This process is mediated by the actin-nucleating Arp2/3 complex acting downstream of WASP/N-WASP, which are known regulators of endocytic processes [25] and are essential for normal immune function [26].

Interestingly, the short-term kinetics of actin flashes (1–2 minutes per flash) are similar for phagosomes containing *Listeria*, latex beads or cryptococci. However, both cryptococcal and latex bead phagosomes also exhibit longer flashes (tens of minutes) that are not seen on *Listeria* phagosomes, presumably because *Listeria* begin to disrupt the phagosome membrane within 30 minutes of invasion [27] and have escaped into the cytoplasm within two hours [28]. Similarly, on phagosomes containing latex beads, actin flashes are essentially absent after 2 hours [16]. In contrast, actin flashes on phagosomes containing *Cryptococcus* could occur over the whole 18 hours of observation indicating either a difference in timing, if the stimulus was identical, or a distinct stimulus for actin flashing on phagosomes containing *Cryptococcus*.

For both *Listeria* and *Cryptococcus* containing phagosomes, the stimulus for actin flashing may be associated with membrane permeabilisation ([15] and our data). However, the stimulus for actin flashing on latex bead containing phagosomes is unknown. It is possible that even these ‘inert’ phagosomes occasionally acquire membrane lesions, which may potentially explain why we observe infrequent actin flashes on heat killed cryptococci. In addition, Liebl and Griffiths have shown that actin flashes on latex bead phagosomes are closely connected with the process of phagosome maturation [16]. Phagosome maturation is a complex process through which the phagosome becomes highly anti-microbial, via fusion with several distinct parts of the endocytic pathway [29]. The correct ordering of these fusion events is essential and thus actin flashes may additionally be important in regulating these vesicle fusion events during normal maturation of the cryptococcal phagosome with live and dead cryptococci. However, in the case of live *Cryptococcus* there is clearly an additional role for actin flashes, since, unlike latex beads, intracellular cryptococci continue to undergo actin flashing within a fully mature phagolysosome and at a much higher incidence than when heat killed.

What, then, is the role of actin flashing on cryptococcal phagosomes? The increased frequency of flashing on phagosomes with live cryptococci suggests a potential link with expulsion, since this occurs frequently with live cryptococci but never with heat-killed cryptococci or latex beads [4], [5]. Imaging of actin dynamics prior to and during expulsion shows that the polymerisation of actin did not provide the driving force for expulsion in agreement with the previous finding that inhibition of actin polymerisation increases, rather than inhibits, expulsion [4], [5]. Instead, we favour a model in which actin flashes act to inhibit cryptococcal expulsion. This is supported by the observation that phagosomes that are eventually expelled (and are thus presumably ‘attempting’ to undergo expulsion) are coated with actin for a greater proportion of their lifetime and the fact that stabilizing actin filaments with jasplakinolide specifically lengthens individual actin flashes and concurrently inhibits cryptococcal escape. The most obvious mechanism suggested by these data is a steric inhibition of membrane fusion by filamentous actin, although we note that other actin regulatory events may be involved in this process as discussed below.

The role of actin dynamics in vesicle exocytosis is highly dependent on the method of actin modulation and the vesicle and cell type. Actin depolymerisation promotes secretion from mast cells [30] and the release of secretory granules (but not secretory vesicles) in neutrophils [31], whereas perturbing actin dynamics abolishes granule exocytosis from cytotoxic T-lymphocytes [32]. This differential role of actin may, in some cases, be due to an alteration in signaling rather than perturbation of the cytoskeleton directly, since treatment of cytotoxic T lymphocytes with either jasplakinolide or latrunculin A abolished granule exocytosis but this block could be bypassed by stimulating granule exocytosis via an alternative pathway [32]. We note, however, that the drug concentrations we have used are at least 10-fold lower, and in many cases 100-fold lower, than in the studies described above, suggesting that actin flashes on cryptococcal phagosomes are exquisitely sensitive to actin perturbation under conditions in which cortical actin and constitutive vesicle trafficking are unaffected.

Using fluorescent dextran as a probe for phagosome membrane integrity we have shown that loss of dextran from the phagosome occurs simultaneously with expulsion, implying that expulsion occurs through fusion of the phagosome with the plasma membrane. Earlier work has provided evidence for permeabilisation of phagosomes containing cryptococci [21], in support of our finding that >90% of phagosomes containing cryptococci are permeabilised within three hours of phagocytosis. Interestingly, permeabilisation appears to be a prerequisite for actin flashing, since flashes are never observed on phagosomes containing dextran.

Taken together, these data suggest a potential model for actin flashing in which permeabilisation of the cryptococcal phagosome induces a signal that results in localized WASp-Arp2/3 activation and subsequent actin polymerization around the phagosome membrane that transiently inhibits cryptococcal expulsion. Elucidating the molecular signals that initiate this process and where actin flashes may be sufficient to block expulsion should provide significant insights into host-pathogen signaling events during intracellular parasitism by cryptococci and, potentially, other infectious organisms.

## Materials and Methods

### *Cryptococcus* culture and opsonisation

Unless otherwise stated all reagents were purchased from Sigma (Poole, UK). Cryptococcal strains were grown for 18 hours overnight in YPD medium (2% glucose, 1% peptone, and 1% yeast extract) with shaking (240 rpm) at 25°C. Yeast from overnight cultures were counted, collected from culture by centrifugation at 6,500 rpm for 2 min and resuspended in PBS at the required concentration. Where required, yeast were heat killed at 55°C for 30 minutes. Yeast were opsonised with 18B7 (a monoclonal antibody to glucuronoxylomannan, a component of the cryptococcal capsule, kindly provided by Arturo Casadevall) for 1 hour at 37°C. Unless otherwise stated strain ATCC90112 was used for all experiments due to its high expulsion and flashing rates.

### Macrophage cell line culture and phagocytosis conditions

J774A.1 macrophages and Raw macrophages stably expressing GFP tagged actin (Raw^GFPactin^, a gift from Dave Knecht) were cultured in Dulbeccos's modified Eagles medium (DMEM) with 10% foetal calf serum (Gibco), 50 U/ml penicillin and 50 µg/ml streptomycin at 37°C in a humidified atmosphere of 5% CO_2_. J774 (1×10^5^) were plated on 13 mm diameter glass cover slips in 24-well plates 18 hours before phagocytosis of cryptococci. J774 were activated with PMA for 1 hour before opsonised cryptococci were added in DMEM for 2 hours at 37°C. Raw^GFPactin^ cells were plated at 1×10^5^ per well in 96-well imaging plate (BD Biosciences) or in glass bottomed 96-well plate (MatTek, Ashland, MA) or at 2.8×10^5^ on 22 mm diameter glass cover slips with 400 U/ml of interferon gamma (ImmunoTools) 18 hours before phagocytosis of cryptococci. Opsonised cryptococci in DMEM were added to Raw^GFPactin^ cells for 2 hours at 37°C. 1 mg/ml TRITC or FITC labelled dextran (70 Kd) was added to opsonised cryptococci during phagocytosis to label phagosomes. IgG coated 3 µm latex beads (prepared as described previously May et al., 2000) were added to macrophages in DMEM for 20 minutes at 37°C.

### Human primary macrophage isolation, differentiation and phagocytosis conditions

Human monocytes were isolated from buffy coats (National Blood Service, Birmingham). Buffy coats were diluted 1∶1 with sterile PBS, layered onto a Ficoll Plaque (GE Healthcare, Vienna) cushion at a ratio of 2∶3 and centrifuged at 2000×g for 30 minutes with no braking. White blood cells were harvested from the Ficoll interface, diluted 1∶1 with sterile PBS and and centrifuged at 1500×g for 5 minutes to pellet cells. The pellet was resuspended in PBS and centrifuged at 1000×g for 5 minutes. This step was repeated until the supernatant was clear, indicating removal of platelets. After the final wash, the cell pellet was resuspended in RPMI 1640 with 2% FBS and counted. 1×10^6^ cells per ml were added to tissue culture flasks and incubated at 37°C in a humidified atmosphere of 5% CO_2_. After 1 hour, non-adherent cells were removed by washing with warm PBS and RPMI 1640 with 10% FBS and100 U/ml granulocyte-macrophage colony-stimulating factor (GM-CSF) was added for differentiation of monocytes to macrophages. Media was replaced after three days and then every two days thereafter. Primary macrophages between three and seven days post differentiation were used for experiments. Primary macrophages were plated at 1.5×10^6^ cells per ml in 24-well plates. Opsonised cryptococci in RPMI 1640 were added to primary macrophages for 2 hours at 37°C.

### Human primary macrophage transformation

Before transformation macrophages were washed once in PBS and then trypsinised with Trypsin/EDTA solution for 30 minutes at room temperature. Detached cells were resuspended in RPMI 1640 with 10% FBS. 5×10^5^ cells were transformed with 2 µg LifeAct GFP (a gift from Roland Wedlich-Soeldner) DNA using the human macrophage nucleofector kit (Amaxa (Lonza), Cologne). After electroporation 0.5ml of RPMI 1640 with 10% FBS was added to the cuvette and the cell suspension removed and added to a further 1ml of RPMI 1640 with 10% FBS. Transformed macrophages were plated at 1.2×10^6^ cells/well in glass bottomed 96-well plates (MatTek, Ashland, MA) or at 2×10^6^ cells/well in glass bottomed 24-well plates (MatTek, Ashland, MA). Macrophages were transformed 24 hours prior to phagocytosis of cryptococci. Opsonised cryptococci in RPMI 1640 were added to transformed primary macrophages for 2 hours at 37°C.

### Drug treatments

Wiskostatin (Calbiochem) was serially diluted from a 1mM stock solution in DMSO into DMEM. Cytochalasin D (Sigma) was diluted from a 1mM stock in DMSO to 100nM in DMEM. Jasplakinolide (a gift from Noni Franklin-Tong, Birmingham) was diluted from a 1mM stock in DMSO to 50nM in DMEM. All drugs were added to cells after phagocytosis of cryptococci to avoid inhibition of uptake.

### Transferrin exocytosis assay

J774 macrophages were pulsed with 10µg/ml Alexa Fluor 568 transferrin (a gift from Josh Rappoport, Birmingham) in DMEM for 30 minutes and then chased with DMEM alone. At 0, 5, 15, 30 and 45 minutes cells were transferred to ice for 30 minutes to stop exocytosis and cause detachment. Cells were fixed in 4% formaldehyde in PBS and fluorescence measured by flow cytometry (FACSCaliber, BD Biosciences). Exocytosis was measured by calculating the geometric mean for each time point and normalising this value to time zero.

### Live imaging of Raw^GFPactin^, J774 and human primary macrophage

After phagocytosis of cryptococci, Raw^GFPactin^ cells and human primary macrophages were washed at least three times and imaged in DMEM without phenol red. The phagocytosis of cryptococci in the presence of FITC dextran by J774 macrophages was imaged in DMEM without phenol red. Time lapse images were captured on a TE2000 (Nikon) with Digital Sight DS-Qi1MC camera (Nikon), 20× objective (Ph1 PLAN APO), using NIS elements AR software (Nikon). Images were captured every 2 minutes for 18 hours. Confocal time lapse images were captured on a A1-R (Nikon) with 60× objective (CFI Plan Apo TIRF oil 1.49NA) in resonant scanner mode using NIS elements AR software (Nikon). The A1-R Z-axis was driven with a piezo drive (Mad City labs, Madison, WI). Both microscopes were enclosed in a temperature controlled and humidified environmental chamber (Okolabs) with 5% CO_2_ at 37°C.

### Labelling and imaging of fixed cells

Cells were fixed for 10 minutes in 4% formaldehyde in PBS. TRITC-phalloidin labelling of actin and ARPC2 immunofluorescence was performed as described previously [33]. Lamp1 labelling (Rat monoclonal 1D4B; Developmental Studies Hybridoma Bank University of Iowa) was performed as described previously [34]. Extracellular cryptococci were labelled with 18B7 before permeabilisation. Membranes were labelled with 5 µg/ml FM-464 (a lipophilic steryl dye; Invitrogen) in PBS for 30 minutes as a final step before mounting. FITC dextran pulsed cells were labelled, without prior permeabilisation, with TRITC phalloidin overnight at 4°C. A Zeiss Axiovert 135TV with 63× (1.25 NA Plan NEOFLUAR Ph3) was used for scoring fixed cells. Confocal images were captured on a SP2 scanning confocal (Leica) with 63× objective (1.40NA HCX PL APO) or on a A1-R (Nikon) with 60× objective (CFI Plan Apo TIRF oil 1.49NA) as indicated in figure legends.

### Image processing

TE2000 images from NIS elements AR were exported as individual tiff files and transformed into tiff stacks using ImageJ. A1R confocal images were processed in NIS elements AR. SP2 confocal images were exported as individual tiff files, Z projections were made using ImageJ. Using z-stack information from the confocal data file, the number of z-dimension pixels that each z-section represented was calculated (see figure legends for specific values). Using a custom ImageJ macro, this scaling was then used to re-slice images stack in defined direction, save this new stack and, where required, project this stack to form an extended focus image. Except where indicated, confocal images are single slices. All Quicktime movies were made using ImageJ with Mpeg4 compression. Individual images for figures were copied from ImageJ into Photoshop CS3 (Adobe), which was then used to form RGB merges and to adjust contrast. Illustrator CS3 (Adobe) was used to assemble figures and add scale bars, time indexes, arrows etc.

### Analysis

Actin flashes were scored by eye and confirmed with intensity analysis of actin GFP fluorescence. Intensity of actin fluorescence in and around phagosomes was measured by calculating the ratio of two regions of interest 1.5× the area of the phagosome; one around the phagosome and the other at distant (control) region within the cell. NIS elements AR (Nikon) was used for analysis of actin fluorescent intensity alone whereas ImageJ was used for comparison of actin and dextran fluorescent intensity. The rate of actin flashes was calculated as the number of actin flashes divided by the lifetime of the phagosome (defined as either the length of the experiment or time until expulsion of *Cryptococcus*). Length of actin flashes was calculated as the number of time frames during which the flash was observable multiplied by the time between frames. Flashes that lasted a single frame were given a length of a single frame. Actin lifetime is the total time that actin was observed around an individual phagosome. Expulsion of *Cryptococcus* was counted as described previously [5]. Phagosome survival was defined as a phagosome that had not expelled a yeast cell at that time point. Colocalisation was calculated using an ImageJ colocalisation plugin (http://rsb.info.nih.gov/ij/plugins/colocalization.html, Pierre Bourdoncle). Briefly, two pixels are considered as co-localised if their intensities were higher than a defined threshold (in this case 50 in a 8-bit image) and the ratio of the two intensities was greater than 50%.

### Statistical analysis

Unless otherwise stated statistical significance was calculated using the Fisher exact test add-in (http://www.obertfamily.com/software/fisherexact.html) in Microsoft Excel. Linear regression R^2^ value for correlation of expulsion and flashing was calculated in Microsoft Excel. Box plots were produced in Sigma plot (Sigma plot Software Inc.), line = median, box = interquartile range (IQR), bar = 5–95%, dots = outliers. Statistical significance in the individual or total length of actin flashes between samples was calculated using a Mann-Whitney U-test (http://elegans.swmed.edu/~leon/stats/utest.html Leon Avery).

## Supporting Information

Figure S1 Illustration of the frequency of actin flashing on different phagosomes. The four sets of images of flashing phagosomes are representative of the 25th percentile (purple), 50th percentile (orange), 75th percentile (blue) and 95th percentile (green) as shown in figure 1B. Images for the 95th percentile are of a single phagosome but have been split into two columns and staggered. The coloured lines that join images to time axis indicate when in the 18 hour period of observation the presented flash occurred. Red boxes and lines indicate that the flash is continuous between these time points.
(3.34 MB TIF)

Figure S2 Actin flashes occur only on cryptococci that are entirely intracellular. (A) Single slices from confocal z-stack at two different z-planes: z = 2 µm, around a flashing *Cryptococcus*-containing phagosome; z = 8.8 µm. Extracellular cryptococci are labelled with 18B7, a monoclonal antibody to capsular components. (B) Single slices from z-stack re-sliced through y-plane. Extracellular cryptococci are clearly separated from *Cryptococcus* containing phagosomes that are flashing.
(3.03 MB TIF)

Figure S3 Actin flashes are not inhibited by phagosome maturation or stimulated by increasing phagosome burden. (A) Bright-field and wide-field fluorescence images of a J774 macrophage fixed 3 hours post phagocytosis and labelled for the phagosome maturation marker LAMP1 and actin. An actin flash surrounds the LAMP1 positive phagosome. Scale bar 10µm. (B) Average number of flashes that occur on individual phagosomes in cells containing 1,2,3 or 4 *Cryptococcus* containing phagosomes. Brightfield and actin-GFP epifluorescence images of RAW macrophage-like cells stably expressing actin-GFP were captured every 2 minutes for 18 hours post phagocytosis of cryptococci and scored for actin flashes (40 cells over 5 independent experiments). Error bars are twice standard error; P = 0.45 (Single factor ANOVA).
(2.25 MB TIF)

Figure S4 Actin-GFP fluorescence dynamics does not show a consistent pattern prior to or during cryptococcal expulsion. (A–H) Normalised actin-GFP intensity around *Cryptococcus* containing phagosomes. Border between white and red region indicates instant of expulsion. Data are from randomly chosen phagosomes and each represents an independent experiment.
(0.92 MB TIF)

Figure S5 Transferrin exocytosis is not altered in the same way as *Cryptococcus* expulsion following actin perturbation. Exocytosis of transferrin was assayed by pulsing RAW macrophage-like cells stably expressing actin-GFP with fluorescent transferrin and measuring loss of fluorescence due to exocytosis by flow cytometry. Treatment with 50 nM jasplikinolide (A), 100nM cytochalasin, (B) and 100nM wiskostatin (C) resulted in a slight decrease in the rate of transferrin exocytosis relative to untreated cells (D, E) in all cases, even though the drugs show opposing effects on cryptococcal expulsion.
(0.58 MB TIF)

Figure S6 *Cryptococcus* is expelled from macrophages by phagosome emptying. (A) Three dimensional time lapse confocal of *Cryptococcus* phagocytosis by J774 macrophage in the presence of FITC dextran. FITC dextran is taken up into the phagosome as the cryptococcal cell is phagocytosed. Orange (1st) and purple (2nd) indicate the uptake of two separate cryptococcal cells. Scale bar 10 µm. (B) Time lapse phase contrast and actin-GFP epifluorescence images of macrophages with FITC dextran labelled *Cryptococcus*-containing phagosomes. Extracellular yeast do not visibly take up FITC dextran (arrow). FITC dextran labelling of phagosomes is stable over many hours; the slight decrease in signal over time is because of gradual photo-bleaching and dilution due to increased numbers of cryptococci enlarging the phagosome. Times are expressed as hours:minutes. (C) Time lapse phase contrast and actin-GFP epifluorescence images of cryptococcal expulsion, same field of view as panel (B). The FITC dextran signal is suddenly lost from the macrophage and cryptococci are expelled simultaneously. Expelled cryptococci have no visible FITC dextran labelling (arrow identifies a representative expelled cryptococcal cell). Times are expressed as hours:minutes:seconds. Scale bars are 10 µm. (D) Processed confocal (SP2) z-stack of a J774 macrophage fixed 3 hours post phagocytosis of cryptococci and labelled for F-actin and membrane. DIC image is a single confocal slice, z,x images are single slices from z-stack re-sliced through x-plane and fluorescence x,y images are maximum z-projections. Note the absence of macrophage membrane above the flashing phagosome. Scale bar is 5 µm.
(7.61 MB TIF)

Table S1 Pre-treatment of *Cryptococcus* with actin modulating drugs does not affect expulsion from macrophages.
(0.03 MB DOC)

Video S1 Actin-GFP recruitment to *Cryptococcus* containing phagosome. Bright-field (right) and wide-field fluorescence (left) of actin-GFP time lapse. Frames were captured every 120 seconds.
(0.23 MB MOV)

Video S2 Confocal time lapse of actin flash on *Cryptococcus* containing phagosome. Single slice transmitted light (left) and actin-GFP (right) image taken from the z-stack used in Video S3. Frames captured every 30 seconds.
(0.84 MB MOV)

Video S3 Three-dimensional reconstruction of confocal z-stack time lapse of actin flash. Frames captured every 30 seconds.
(0.84 MB MOV)

Video S4 Three-dimensional reconstruction of confocal z-stack time lapse of actin flash in human primary macrophage transiently expressing F-actin probe Lifeact. Frames captured every 30 seconds.
(1.52 MB MOV)

Video S5 Three-dimensional reconstruction of actin flash in J774 macrophage labelled with F-actin binding drug phalloidin. Three-dimensional reconstruction and rotation from confocal (SP2) z-stack.
(0.28 MB MOV)

Video S6 Three-dimensional reconstruction of actin flash in RAW macrophage-like cell stably expressing actin-GFP. Three-dimensional reconstruction and rotation from single time-point of confocal z-stack used in Video S3.
(1.35 MB MOV)

Video S7 Fluorescently labelled dextran is a stable probe for *Cryptococcus* containing phagosomes. Phase contrast and wide field fluorescence of TRITC-dextran time lapse. Frames were captured every 120 seconds.
(0.39 MB MOV)

Video S8 Fluorescently labelled dextran is lost from phagosomes upon expulsion of *Cryptococcus*. Phase contrast and wide field fluorescence of TRITC-dextran time lapse. Frames were captured every 120 seconds.
(0.35 MB MOV)

Video S9 Loss of dextran from cryptococcal phagosomes is directly followed by an actin flash. From left to right phase contrast, wide field fluorescence of TRITC-dextran and wide field fluorescence of actin-GFP. Frames were captured every 120 seconds.
(0.30 MB MOV)
