# Supplementary figures and images for: The Human Fungal Pathogen Cryptococcus neoformans Escapes Macrophages by a Phagosome Emptying Mechanism That Is Inhibited by Arp2/3 Complex-Mediated Actin Polymerisation
Source: PLoS Pathog. 2010 Aug 12;6(8):e1001041. doi: 10.1371/journal.ppat.1001041 (PMC2920849; doi:10.1371/journal.ppat.1001041)

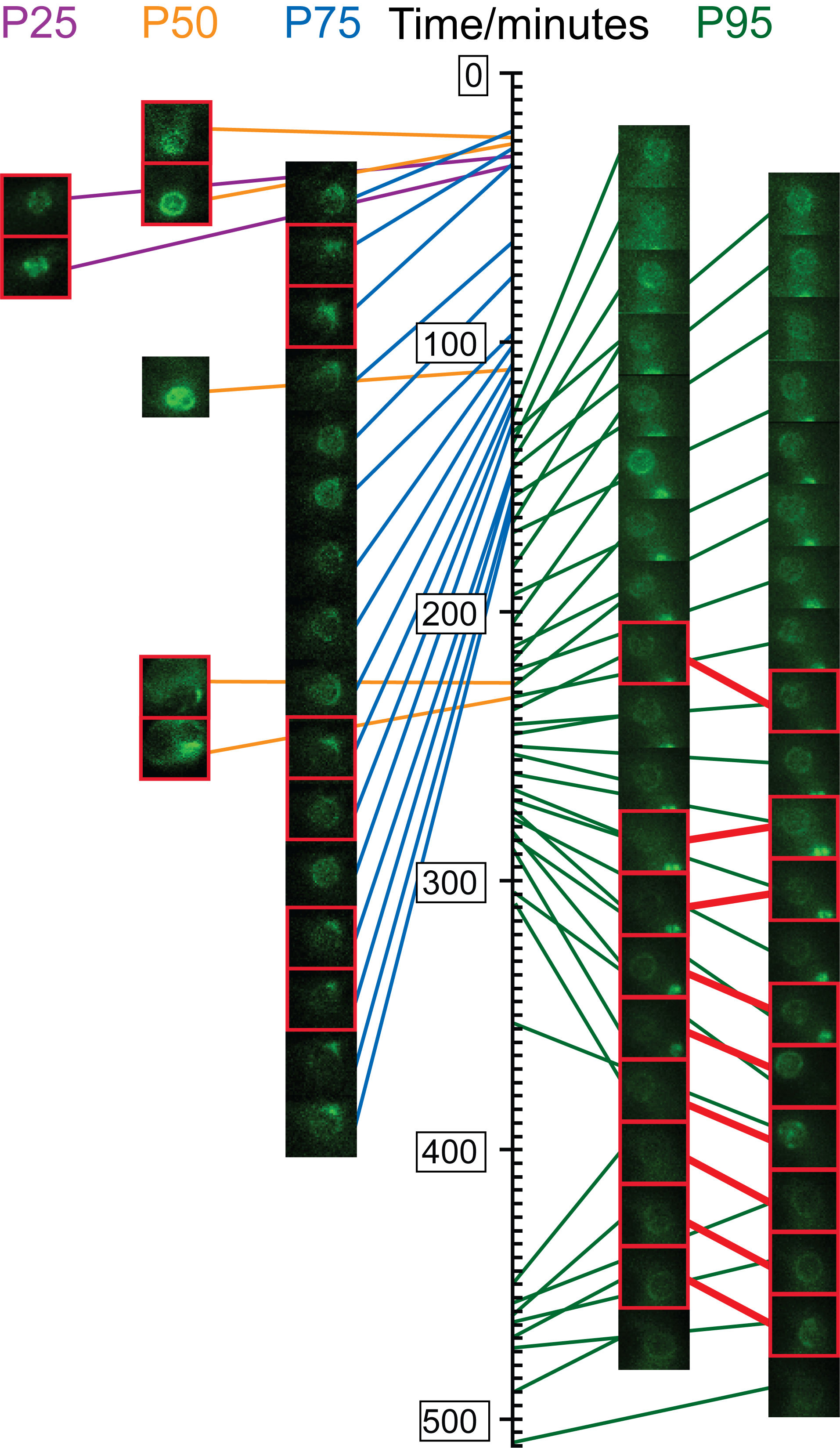

Supplement: Figure S1 — Illustration of the frequency of actin flashing on different phagosomes. The four sets of images of flashing phagosomes are representative of the 25th percentile (purple), 50th percentile (orange), 75th percentile (blue) and 95th percentile (green) as shown in figure 1B. Images for the 95th percentile are of a single phagosome but have been split into two columns and staggered. The coloured lines that join images to time axis indicate when in the 18 hour period of observation the presented flash occurred. Red boxes and lines indicate that the flash is continuous between these time points. (3.34 MB TIF) [file ppat.1001041.s001.tif]

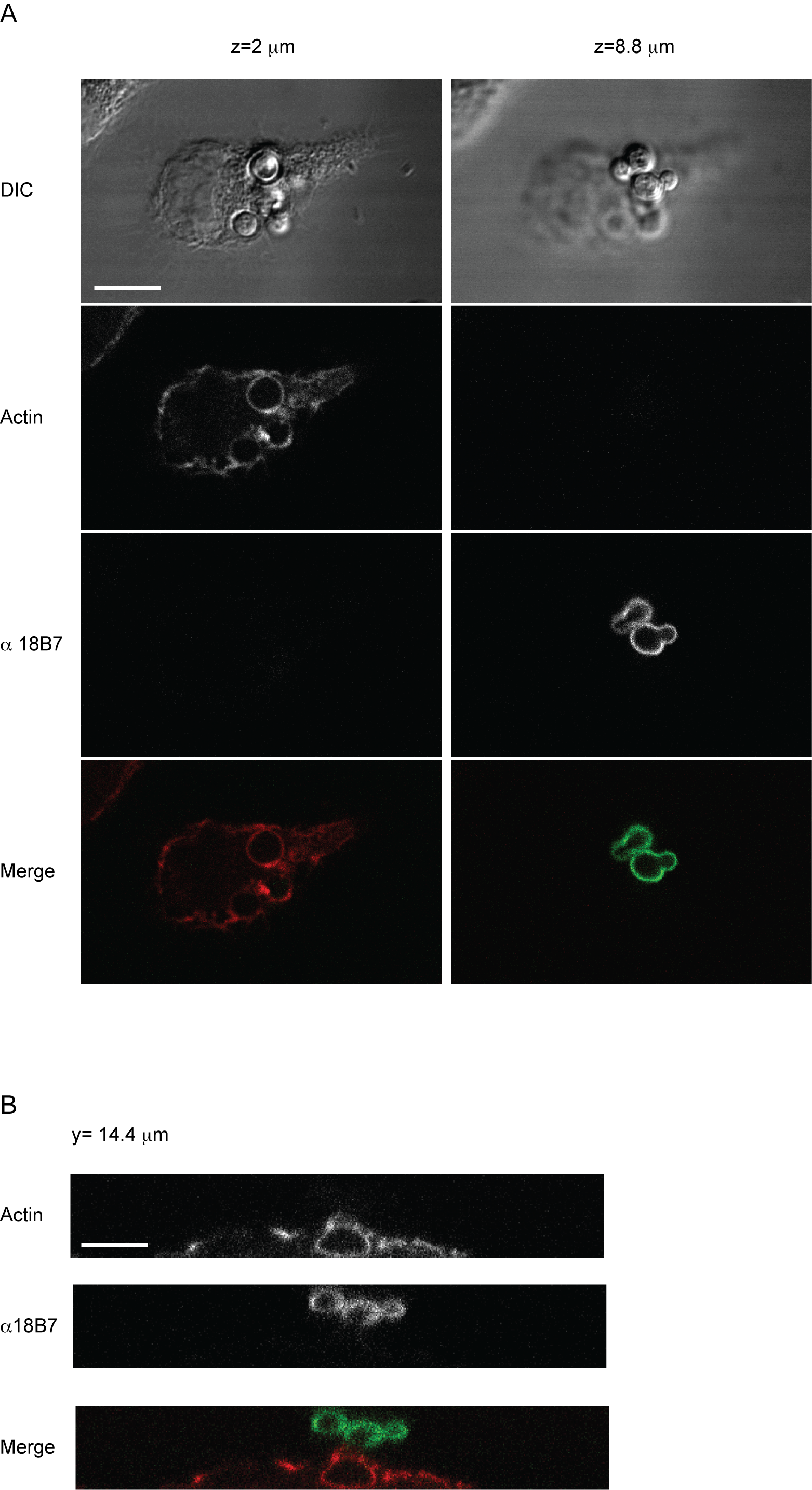

Supplement: Figure S2 — Actin flashes occur only on cryptococci that are entirely intracellular. (A) Single slices from confocal z-stack at two different z-planes: z = 2 µm, around a flashing Cryptococcus-containing phagosome; z = 8.8 µm. Extracellular cryptococci are labelled with 18B7, a monoclonal antibody to capsular components. (B) Single slices from z-stack re-sliced through y-plane. Extracellular cryptococci are clearly separated from Cryptococcus containing phagosomes that are flashing. (3.03 MB TIF) [file ppat.1001041.s002.tif]

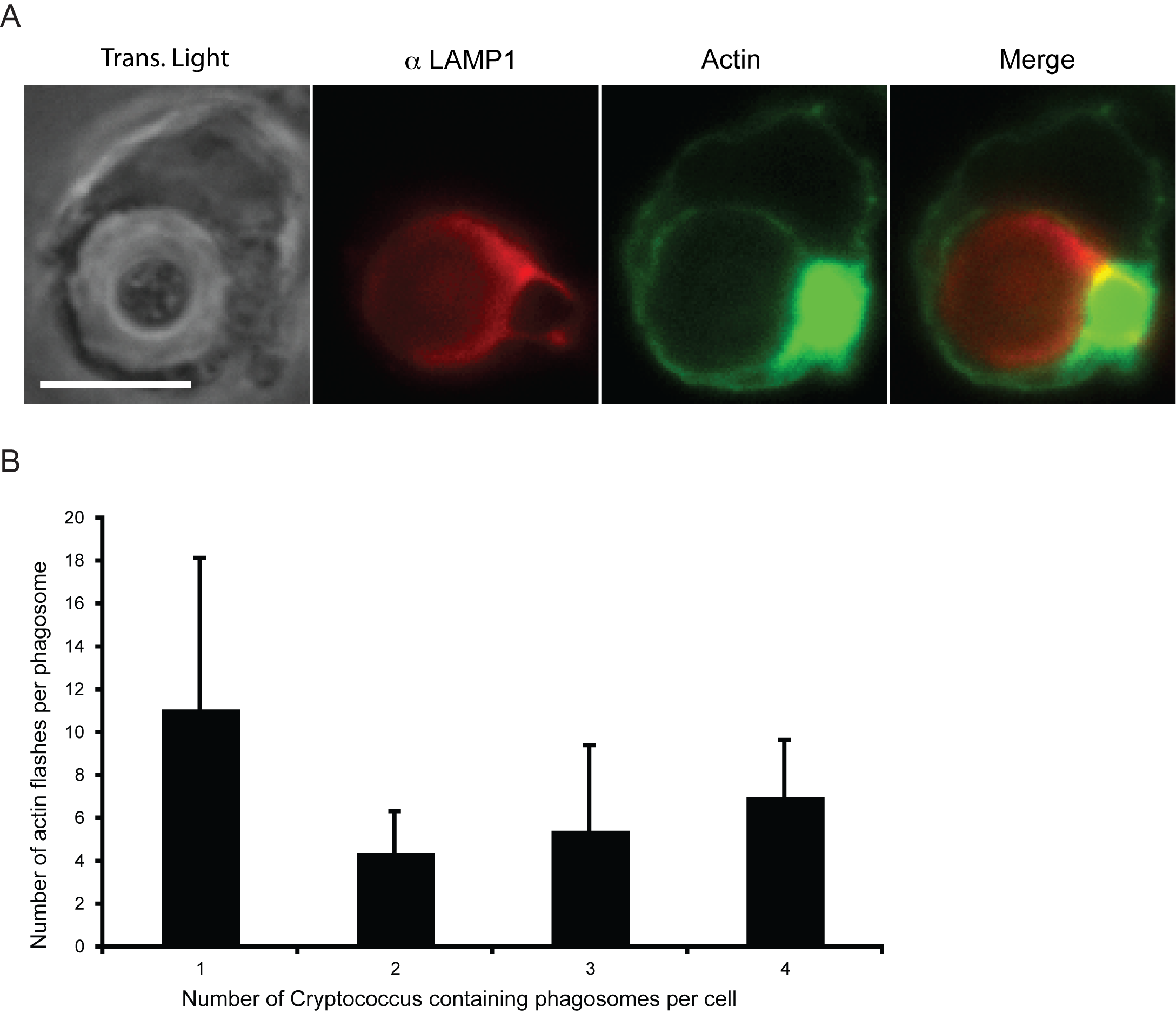

Supplement: Figure S3 — Actin flashes are not inhibited by phagosome maturation or stimulated by increasing phagosome burden. (A) Bright-field and wide-field fluorescence images of a J774 macrophage fixed 3 hours post phagocytosis and labelled for the phagosome maturation marker LAMP1 and actin. An actin flash surrounds the LAMP1 positive phagosome. Scale bar 10µm. (B) Average number of flashes that occur on individual phagosomes in cells containing 1,2,3 or 4 Cryptococcus containing phagosomes. Brightfield and actin-GFP epifluorescence images of RAW macrophage-like cells stably expressing actin-GFP were captured every 2 minutes for 18 hours post phagocytosis of cryptococci and scored for actin flashes (40 cells over 5 independent experiments). Error bars are twice standard error; P = 0.45 (Single factor ANOVA). (2.25 MB TIF) [file ppat.1001041.s003.tif]

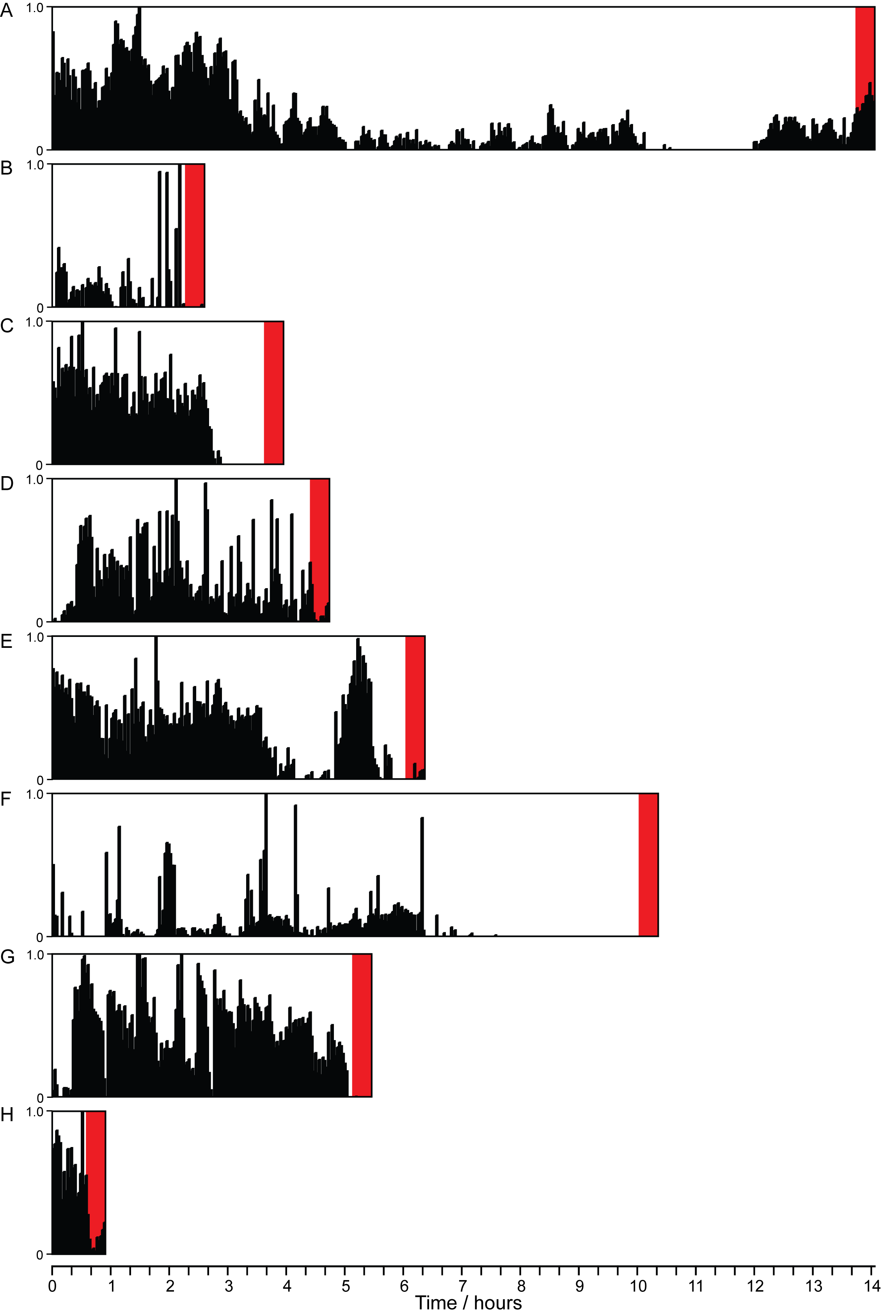

Supplement: Figure S4 — Actin-GFP fluorescence dynamics does not show a consistent pattern prior to or during cryptococcal expulsion. (A–H) Normalised actin-GFP intensity around Cryptococcus containing phagosomes. Border between white and red region indicates instant of expulsion. Data are from randomly chosen phagosomes and each represents an independent experiment. (0.92 MB TIF) [file ppat.1001041.s004.tif]

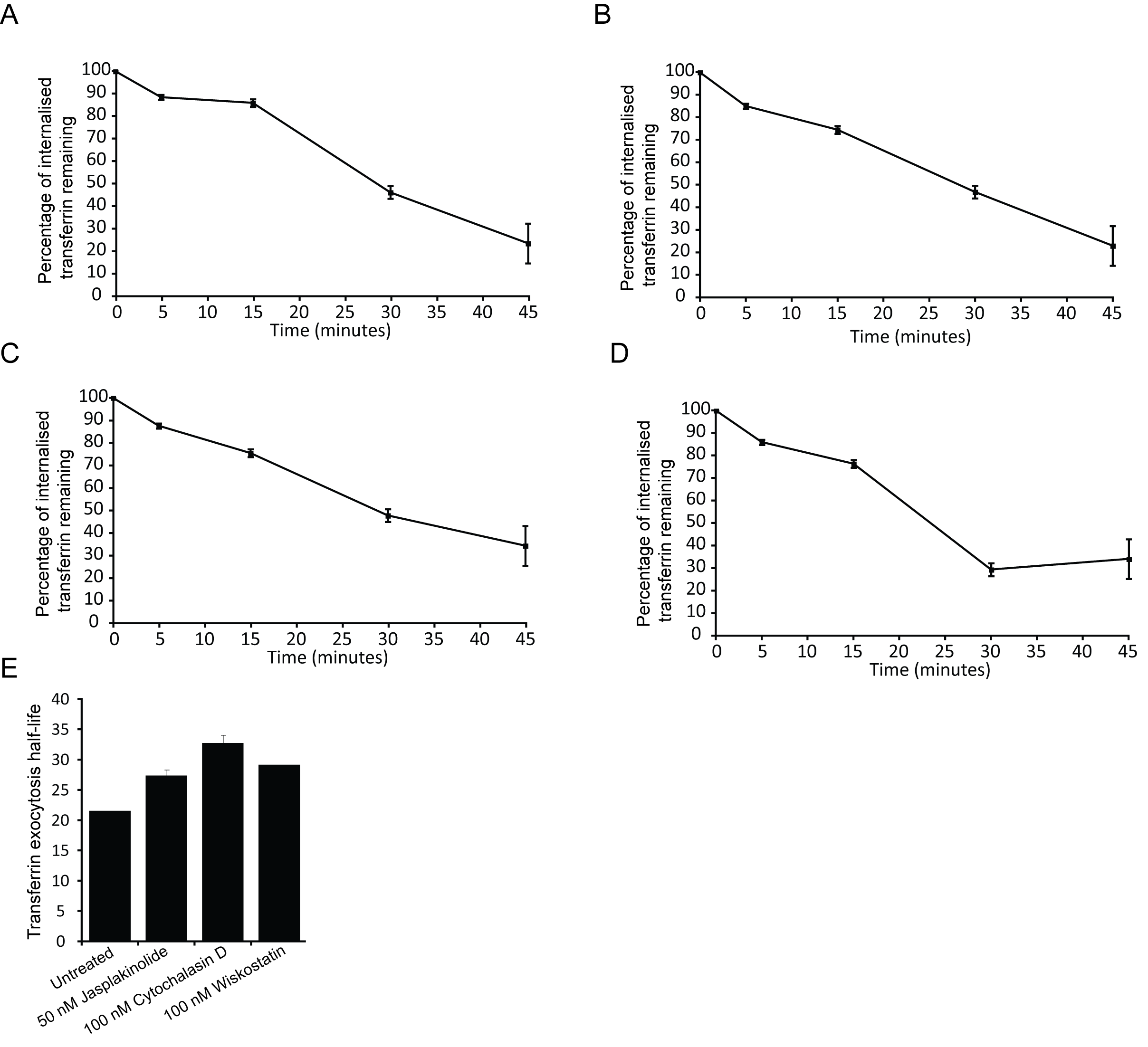

Supplement: Figure S5 — Transferrin exocytosis is not altered in the same way as Cryptococcus expulsion following actin perturbation. Exocytosis of transferrin was assayed by pulsing RAW macrophage-like cells stably expressing actin-GFP with fluorescent transferrin and measuring loss of fluorescence due to exocytosis by flow cytometry. Treatment with 50 nM jasplikinolide (A), 100nM cytochalasin, (B) and 100nM wiskostatin (C) resulted in a slight decrease in the rate of transferrin exocytosis relative to untreated cells (D, E) in all cases, even though the drugs show opposing effects on cryptococcal expulsion. (0.58 MB TIF) [file ppat.1001041.s005.tif]

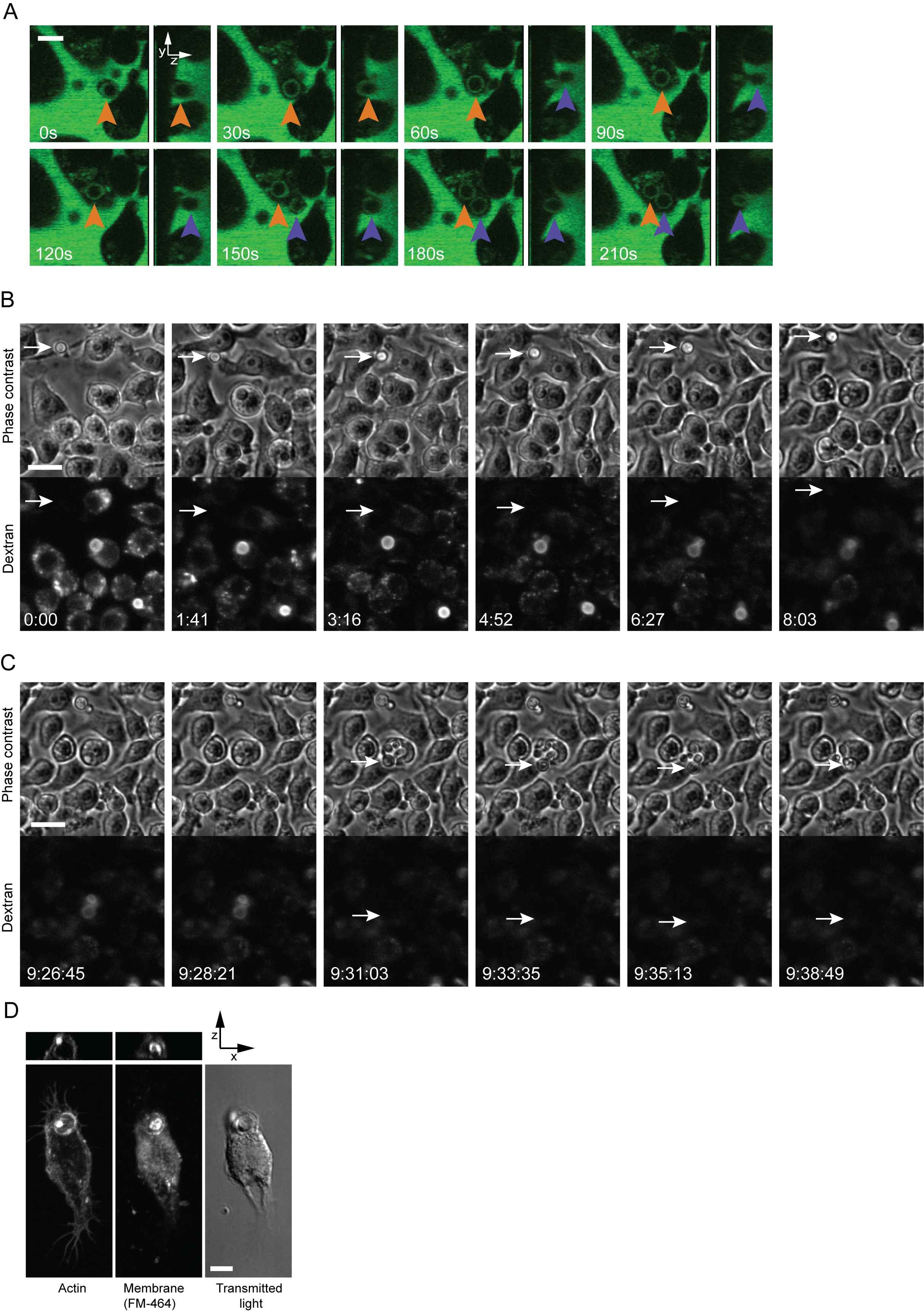

Supplement: Figure S6 — Cryptococcus is expelled from macrophages by phagosome emptying. (A) Three dimensional time lapse confocal of Cryptococcus phagocytosis by J774 macrophage in the presence of FITC dextran. FITC dextran is taken up into the phagosome as the cryptococcal cell is phagocytosed. Orange (1st) and purple (2nd) indicate the uptake of two separate cryptococcal cells. Scale bar 10 µm. (B) Time lapse phase contrast and actin-GFP epifluorescence images of macrophages with FITC dextran labelled Cryptococcus-containing phagosomes. Extracellular yeast do not visibly take up FITC dextran (arrow). FITC dextran labelling of phagosomes is stable over many hours; the slight decrease in signal over time is because of gradual photo-bleaching and dilution due to increased numbers of cryptococci enlarging the phagosome. Times are expressed as hours:minutes. (C) Time lapse phase contrast and actin-GFP epifluorescence images of cryptococcal expulsion, same field of view as panel (B). The FITC dextran signal is suddenly lost from the macrophage and cryptococci are expelled simultaneously. Expelled cryptococci have no visible FITC dextran labelling (arrow identifies a representative expelled cryptococcal cell). Times are expressed as hours:minutes:seconds. Scale bars are 10 µm. (D) Processed confocal (SP2) z-stack of a J774 macrophage fixed 3 hours post phagocytosis of cryptococci and labelled for F-actin and membrane. DIC image is a single confocal slice, z,x images are single slices from z-stack re-sliced through x-plane and fluorescence x,y images are maximum z-projections. Note the absence of macrophage membrane above the flashing phagosome. Scale bar is 5 µm. (7.61 MB TIF) [file ppat.1001041.s006.tif]
