# Supplementary material for: The Human Fungal Pathogen Cryptococcus neoformans Escapes Macrophages by a Phagosome Emptying Mechanism That Is Inhibited by Arp2/3 Complex-Mediated Actin Polymerisation
Source: PLoS Pathog. 2010 Aug 12;6(8):e1001041. doi: 10.1371/journal.ppat.1001041 (PMC2920849; doi:10.1371/journal.ppat.1001041)
Supplement: Table S1 — Pre-treatment of Cryptococcus with actin modulating drugs does not affect expulsion from macrophages. (0.03 MB DOC) [file ppat.1001041.s007.doc]

| **Drug treatment** | **% expelled** | | **Number of cells observed** | |
| --- | --- | --- | --- | --- |
|  | **2 hours** | **18 hours** | **2 hours** | **18 hours** |
| Untreated | 22.9 | 23.0 | 298 | 404 |
| 50nM Jasplakinolide | 21.5 | 22.8 | 330 | 313 |
| 100nM Cytochalasin D | 23.1 | 24.5 | 316 | 374 |
| 100nM Wiskostatin | 23.5 | 22.9 | 388 | 295 |
